# A gene variant near *ATM* is significantly associated with metformin treatment response in type 2 diabetes: a replication and meta-analysis of five cohorts

**DOI:** 10.1007/s00125-012-2537-x

**Published:** 2012-03-28

**Authors:** N. van Leeuwen, G. Nijpels, M. L. Becker, H. Deshmukh, K. Zhou, B. H. C. Stricker, A. G. Uitterlinden, A. Hofman, E. van ’t Riet, C. N. A. Palmer, B. Guigas, P. E. Slagboom, P. Durrington, R. A. Calle, A. Neil, G. Hitman, S. J. Livingstone, H. Colhoun, R. R. Holman, M. I. McCarthy, J. M. Dekker, L. M. ’t Hart, E. R. Pearson

**Affiliations:** 1 Department of Molecular Cell Biology, Leiden University Medical Center, Leiden, the Netherlands; 2 EMGO Institute for Health and Care Research, VU University Medical Center, Amsterdam, the Netherlands; 3 Department of General Practice, VU University Medical Center, Amsterdam, the Netherlands; 4 Department of Epidemiology, Erasmus Medical Center, Rotterdam, the Netherlands; 5 Department of Hospital Pharmacy, Erasmus Medical Center, Rotterdam, the Netherlands; 6 University of Dundee, Medical Research Institute, Dundee, DD1 9SY UK; 7 Department of Epidemiology and Biostatistics, VU University Medical Center, Amsterdam, the Netherlands; 8 Leiden University Medical Center Molecular Epidemiology, Postzone S5-P, PO box 9600, 2300RC Leiden, the Netherlands; 9 Netherlands Consortium for Healthy Ageing, the Netherlands, www.healthy-ageing.nl; 10 University of Manchester, School of Medicine, Manchester, UK; 11 Pfizer Ltd, New York, NY USA; 12 Department of Primary Health Care, University of Oxford, Oxford, UK; 13 Barts and the London School of Medicine and Dentistry, Queen Mary University of London, London, UK; 14 Oxford NIHR Biomedical Research Centre, Churchill Hospital, Oxford, UK; 15 Oxford Centre for Diabetes, Endocrinology and Metabolism, University of Oxford, Churchill Hospital, Oxford, UK; 16 Wellcome Trust Centre for Human Genetics, University of Oxford, Oxford, UK

**Keywords:** Genetics of type 2 diabetes, Human, Meta-analysis, Metformin, Oral pharmacological agents

## Abstract

**Aims/hypothesis:**

In this study we aimed to replicate the previously reported association between the glycaemic response to metformin and the SNP rs11212617 at a locus that includes the ataxia telangiectasia mutated (*ATM*) gene in multiple additional populations.

**Methods:**

Incident users of metformin selected from the Diabetes Care System West-Friesland (DCS, *n* = 929) and the Rotterdam Study (*n* = 182) from the Netherlands, and the CARDS Trial (*n* = 254) from the UK were genotyped for rs11212617 and tested for an association with both HbA_1c_ reduction and treatment success, defined as the ability to reach the treatment target of an HbA_1c_ ≤7 % (53 mmol/mol). Finally, a meta-analysis including data from literature was performed.

**Results:**

In the DCS cohort, we observed an association between rs11212617 genotype and treatment success on metformin (OR 1.27, 95% CI 1.03, 1.58, *p* = 0.028); in the smaller Rotterdam Study cohort, a numerically similar but non-significant trend was observed (OR 1.45, 95% CI 0.87, 2.39, *p* = 0.15); while in the CARDS cohort there was no significant association. In meta-analyses of these three cohorts separately or combined with the previously published cohorts, rs11212617 genotype is associated with metformin treatment success (OR 1.24, 95% CI 1.04, 1.49, *p* = 0.016 and OR 1.25, 95% CI 1.33, 1.38, *p* = 7.8 × 10^−6^, respectively).

**Conclusions/interpretation:**

A gene variant near *ATM* is significantly associated with metformin treatment response in type 2 diabetic patients from the Netherlands and the UK. This is the first robustly replicated common susceptibility locus found to be associated with metformin treatment response.

**Electronic supplementary material:**

The online version of this article (doi:10.1007/s00125-012-2537-x) contains peer-reviewed but unedited supplementary material, which is available to authorised users.

## Introduction

Metformin is the first-choice treatment for type 2 diabetes [1]. It mainly improves fasting glucose levels by decreasing hepatic glucose production by inhibiting the mitochondrial respiratory-chain complex [2, 3], leading to activation of the AMP-activated protein kinase (AMPK) [4] through increased cellular AMP [5]. Activation of the AMPK pathways was thought to be an important underlying mechanism involved in the inhibition of hepatic gluconeogenesis by metformin [6]; however, AMPK-independent glucose-lowering effects of metformin have been described recently [7], and therefore the exact molecular mechanism of action remains unclear.

Our recent genome-wide association study revealed that the single-nucleotide polymorphism (SNP) rs11212617, located near the ataxia telangiectasia mutated (*ATM*) gene, is associated with metformin treatment response [8]. It has been shown in UK participants with type 2 diabetes mellitus from the GoDARTS (Genetics of Diabetes Audit and Research Tayside) and UKPDS (UK Prospective Diabetes Study) cohorts (*n* = 3,920) that the minor C allele of rs11212617 is associated with two successful treatment outcomes: (1) the ability to achieve HbA_1c_ values ≤7% (53 mmol/mol); (2) lower HbA_1c_ when analysed as a quantitative trait [8]. rs11212617 is located in a large linkage disequilibrium (LD) block that includes other genes as well; however, *ATM* is the most likely candidate to be involved [9, 10].

In the Diabetes Prevention Program (DPP, *n* = 2,981), there was no evidence of an association between rs11212617 and the effect of metformin in delaying disease progression from impaired glucose tolerance to diabetes (CC vs AA, HR 1.22) [95% CI 0.86, 1.74] *p* = 0.27) [11]. Lack of replication is often caused by the use of different phenotypes. As the progression to diabetes phenotype differs markedly from the type 2 diabetes mellitus phenotype, we have attempted to replicate our original finding in three independent cohorts using the identical phenotypic definition to that used in the original discovery. For this study, we used the Diabetes Care System West-Friesland (DCS) [12] and the Rotterdam Study [13] from the Netherlands, and the multi-centre CARDS (Collaborative Atorvastatin Diabetes Study) Trial [14] from the UK. Data were analysed in the individual cohorts, separately and combined in a meta-analysis. Finally an additional meta-analysis was performed using these new cohorts combined with the previously described GoDARTS and UKPDS stage 2 replication cohorts [8].

## Methods

### Study cohorts and participants

Inclusion criteria for all cohorts were: white ethnicity; continuous metformin treatment for at least 6 months; a pretreatment HbA_1c_ value measured within 6 months of the start of metformin and at least one measurement within the 18 months after; no other glucose-lowering medication prescription except stable sulfonylurea treatment before and during metformin treatment. Ethics approval was obtained from the Medical Ethics Committee of the VU Vrije Universiteit Medical Center for the DCS, the Erasmus Medical Center for the Rotterdam Study, and for CARDS each centre obtained local research ethics committee approval after approval from the Multi-centre Research Ethics Committee. All participants gave written informed consent.

### Diabetes Care System West-Friesland

The DCS started in 1996 and is a diabetes management model which aims to improve diabetes care by coordinating the different types of diabetes care and to improve patient empowerment by providing education. Patients from the West-Friesland region in the Netherlands visit a local DCS centre once a year for a medical examination; therefore longitudinal information on medical history, drug use and drug response is available for all patients (*n* = 5,424) [12]. Metformin response could be defined in 929 white patients, and their characteristics are shown in Table 1.

**Table 1:** Characteristics of the cohorts included in this study

Characteristic	DCS	Rotterdam Study	CARDS
*n*	929	182	254
Age (years)	63.4 ± 10.0	74.2 ± 8.1	61.4 ± 8.8
Male (%)	56.4	45.1	69.0
BMI	30.1 ± 4.9	NA	NA
Baseline HbA_1c_ (%)	6.7 ± 1.0	8.3 ± 1.5	8.7 ± 1.4
Baseline HbA_1c_ (mmol/mol)	50 ± 11	67 ± 16	72 ± 15
Adherence	NA	88.4 ± 14.7	NA
eGFR	91.8 ± 36.1	NA	NA
Responders (%)	67.1	57.7	52.0
Metformin monotherapy (%)	58.8	35.7	31.0
Genotype frequency rs11212617; AA/AC/CC (%)	32.5/47.1/20.4	32.4/46.7/20.9	32.3/44.4/23.2
Quality^a^ (selection, comparability, exposure)	****, *^b^, ***	****,*^b^, ***	****,*^b^, ***

Data are means±SD or *n* (%)

^a^Quality of the studies assessed with the Newcastle–Ottawa Assessment Scale (www.ohri.ca/programs/clinical_epidemiology/oxford.asp); the maximum scores for selection, comparability and exposure are ****, ** and ***, respectively. GoDARTS and UKPS are not represented in this table and both scored the maximum of ****, ** and ***

^b^The Rotterdam Study and CARDS lacked the covariates BMI and eGFR in the analysis, and the DCS lacked the covariate adherence in the analysis, therefore these studies only scored one star for comparability

eGFR, estimated glomular filtration rate calculated with the Cockcroft–Gault formula; NA, not available

Patients were genotyped for rs11212617 with a Taqman assay (Assay ID C_1314213; Applied Biosystems, Nieuwerkerk a/d IJssel, the Netherlands) according to the manufacturer’s protocol. The call rate was 97.6%, and there was no deviation from Hardy–Weinberg equilibrium (HWE; *p* = 0.46).

### Rotterdam Study

The Rotterdam Study is a prospective population-based cohort study of 10,994 whites aged 55 years and older in Rotterdam, the Netherlands. Patients were invited between 1990 and 1999 and have been followed since then. The aim of the study was to investigate determinants of chronic and disabling diseases [13]. Medication prescription data were available for nearly all participants. Metformin response could be defined in 182 patients, and their characteristics are shown in Table 1.

Participants were genotyped for rs609261, which is in high LD with rs11212617 (*r* ^2^ = 1.0, HAPMAP, CEU) using the Illumina 550 k SNP array according to the manufacturer’s instructions. Quality controls were as described previously [15]. The call rate was 99.9%, and there was no deviation from HWE (*p* = 0.75).

### CARDS

CARDS is a multicentre, placebo-controlled, double-blind study that enrolled 2,838 white men and women between 40 and 75 years of age and randomised them to receive 10 mg/day of atorvastatin or placebo [14]. Patients had type 2 diabetes as well as one other risk factor for coronary heart disease. CARDS was carried out in 132 centres in the UK and Ireland. Metformin response as defined above could be characterised in 254 patients, and their characteristics are shown in Table 1.

DNA was genotyped at Perlegen Sciences (Mountain View, CA, USA) using a proprietary SNP set comprising 599164 SNPs. The call rate for rs11212617 was 99%, and there was no deviation from HWE (*p* = 0.1).

### Statistical analysis

Two different outcomes were used to measure the metformin treatment response: (1) the ability to reach the treatment target of HbA_1c_ ≤7 % (53 mmol/mol), analysed with logistic regression; (2) the decrease in HbA_1c_ achieved, analysed as a quantitative trait with linear regression. In each cohort, the total group of metformin users was analysed, but also a separate analysis was performed on patients starting metformin monotherapy or those starting dual therapy (where metformin is added to stable sulfonylurea treatment). Furthermore, two separate analyses were performed, the first on the total patient group regardless of the baseline HbA_1c_, and the second using only individuals with a baseline HbA_1c_ >7 % (53 mmol/mol). The definition of the endpoints and covariates was similar to the original publication [8]. The covariates that were included in both the logistic and linear regression analyses were: baseline HbA_1c_, time between baseline HbA_1c_ and metformin start date, drug adherence, daily dose and creatinine clearance, if measured in the cohort. In all cohorts with genome-wide association study data available, principal component analysis was used to exclude participants of non-white origin. A priori power calculations using Quanto software (http://hydra.usc.edu/gxe) based on the ORs obtained in our original publication (1.35 or 1.25 when only stage 2 replication data are included) showed that we had respectively 86% and 61% power in the DCS, 29% and 18% in the Rotterdam Study, and 39% and 24% in CARDS (*α* = 0.05). Data were considered to be significant at *p* < 0.05.

### Meta-analysis

The three cohorts described above were included in the meta-analyses of treatment success and treatment HbA_1c_. To assess the overall robustness of the association, a second set of meta-analyses was performed by also including the stage 2 replication cohorts previously described by Zhou et al: GoDARTS (*n* = 1,965) and UKPDS (*n* = 1,113). To avoid an overestimation of the effect of the SNP, the initial GoDARTS discovery cohort, which showed a very strong and highly significant association with treatment success (OR 1.64; 95% CI 1.37, 1.99), was not included. The DPP Study was not included because of the substantial nature of the difference in the phenotype [11].

For meta-analysis, a fixed-effects model was used. The inconsistency index *I* ^2^ was used to assess between-study heterogeneity. To assess bias, funnel plots were generated, and Egger and Harbord tests were performed for, respectively, the linear and the logistic model. The meta-analysis was performed using Comprehensive Meta-analysis software (Biostat, Englewood, NJ, USA) and Stata software version 11.2 (Stata, College Station, TX, USA).

## Results

The minor allele and genotype frequencies of the SNP, rs11212617, were similar in the three new cohorts (*p* > 0.2) and consistent with those reported for the GoDARTS and UKPDS cohorts. The minor allele frequency was 0.44, and the genotype frequencies are shown in Table 1.

In the DCS cohort, including both patients receiving monotherapy and patients receiving dual therapy, we observed, as in our original publication, an association between the minor C allele of rs11212617 and treatment success on metformin (OR 1.27 [95% 1.03, 1.58], *p* = 0.028; Table 2). In the Rotterdam Study, we observed a similar but non-significant trend (OR 1.45 [95% CI 0.87, 2.39], *p* = 0.15), but this was not seen in the CARDS cohort (OR 1.03 [95% CI 0.68, 1.57], *p* = 0.86). In all three cohorts, the effect of rs11212617 was larger in those receiving monotherapy compared with dual therapy (Table 2). In the analysis of on-treatment HbA_1c_ as a quantitative trait, there was no significant effect on HbA_1c_ in either the DCS or the Rotterdam Study cohort. The only significant association was observed in the dual therapy group of the CARDS cohort, where the C allele was associated with higher-treatment HbA_1c_ (Table 3). There was no association between rs11212617 genotype and baseline HbA_1c_ in any of the cohorts (data not shown).

**Table 2:** Logistic regression for the ability to reach the metformin treatment target of HbA_1c_ <7 % (53 mmol/mol) according to the *ATM* rs11212617 genotype

Study	Group	*n*	OR (95% CI)	SE	*p* value
DCS	Total group	929	1.27 (1.03, 1.58)	0.14	0.028
	Monotherapy	547	1.32 (0.99, 1.78)	0.19	0.062
	Dual therapy	382	1.20 (0.87, 1.66)	0.19	0.26
Rotterdam Study	Total group	182	1.44 (0.87, 2.39)	0.36	0.15
	Monotherapy	65	1.97 (0.72, 5.42)	0.19	0.19
	Dual therapy	117	1.40 (0.77, 2.57)	0.30	0.27
CARDS	Total group	254	1.03 (0.68, 1.57)	0.21	0.86
	Monotherapy	81	1.50 (0.76, 2.95)	0.34	0.23
	Dual therapy	173	0.82 (0.46, 1.46)	0.29	0.51
Meta-analysis	Total group	1,365	1.24 (1.04, 1.49)	0.09	0.016
	Monotherapy	693	1.38 (1.07, 1.80)	0.13	0.015
	Dual therapy	672	1.15 (0.89, 1.48)	0.13	0.29
Meta-analysis including stage 2 replication cohorts previously used by Zhou et al [8]					
GoDARTS	Total group	1,965	1.21 (1.05, 1.38)	0.07	0.008
	Monotherapy	1,460	1.25 (1.07, 1.46)	0.08	0.005
	Dual therapy	505	1.08 (0.84, 1.40)	0.13	0.54
UKPDS	Total group	1,113	1.37 (1.10, 1.72)	0.11	0.006
	Monotherapy	284	1.82 (1.20, 2.78)	0.21	0.005
	Dual therapy	829	1.23 (0.94, 1.62)	0.14	0.13
Meta-analysis	Total group	4,443	1.25 (1.13, 1.38)	0.05	7.8 × 10^−6^
	Monotherapy	2,437	1.33 (1.16, 1.50)	0.07	1.4 × 10^−5^
	Dual therapy	2,006	1.15 (0.99, 1.34)	0.08	0.067

Patients are included regardless of their baseline HbA_1c_. Additive logistic regression models were used to calculate the C-allelic OR in each cohort. Covariates included were baseline HbA_1c_, baseline gap (except DCS), daily dose, drug adherence (except for DCS and CARDS), and eGFR (except for CARDS and Rotterdam Study). In the meta-analysis, a fixed-effects model was used

**Table 3:** Linear regression for treatment HbA_1c_ per *ATM* allele

Study	Group	*n*	Beta (95% CI)	SE	*p* value
DCS	Total group	929	−0.020 (−0.095, 0.054)	0.038	0.59
	Monotherapy	547	0.026 (−0.065, 0.117)	0.046	0.58
	Dual therapy	382	−0.082 (−0.209, 0.045)	0.065	0.21
Rotterdam Study	Total group	182	−0.053 (−0.205, 0.098)	0.077	0.49
	Monotherapy	65	−0.107 (−0.327, 0.113)	0.112	0.33
	Dual therapy	117	−0.069 (−0.266, 0.127)	0.100	0.49
CARDS	Total group	254	0.163 (−0.031, 0.334)	0.082	0.06
	Monotherapy	81	−0.091 (−0.414, 0.213)	0.160	0.54
	Dual therapy	173	0.286 (0.089, 0.476)	0.100	0.005
Meta-analysis	Total group	1,365	0.001 (−0.061, 0.062)	0.032	0.99
	Monotherapy	693	−0.000 (−0.082, 0.081)	0.041	0.99
	Dual therapy	672	0.004 (−0.089, 0.098)	0.048	0.92
Meta-analysis including stage 2 replication cohorts previously used by Zhou et al [8]					
GoDARTS	Total group	1,965	−0.071 (−0.129, −0.014)	0.030	0.016
	Monotherapy	1,460	−0.080 (−0.147, −0.013)	0.034	0.020
	Dual therapy	505	−0.060 (−0.170, 0.050)	0.057	0.29
UKPDS	Total group	1,113	−0.123 (−0.228, −0.019)	0.053	0.021
	Monotherapy	284	−0.286 (−0.465, −0.107)	0.091	0.001
	Dual therapy	829	−0.067 (−0.193, 0.058)	0.064	0.30
Meta-analysis	Total group	4,443	−0.050 (−0.089, −0.010)	0.020	0.013
	Monotherapy	2,437	−0.066 (−0.116, −0.017)	0.025	0.009
	Dual therapy	2,006	−0.033 (−0.095, 0.029)	0.001	0.29

Patients are included regardless of their baseline HbA_1c_. An additive linear regression model was used to calculate the per C allele change in treatment HbA_1c_ (%). Covariates included were baseline HbA_1c_, baseline gap (except DCS), daily dose, drug adherence (except for DCS and CARDS), and eGFR (except for CARDS and Rotterdam Study). The *p* value of the genotype effect is given. In the meta-analysis, a fixed-effects model was used

**Fig. 1:**
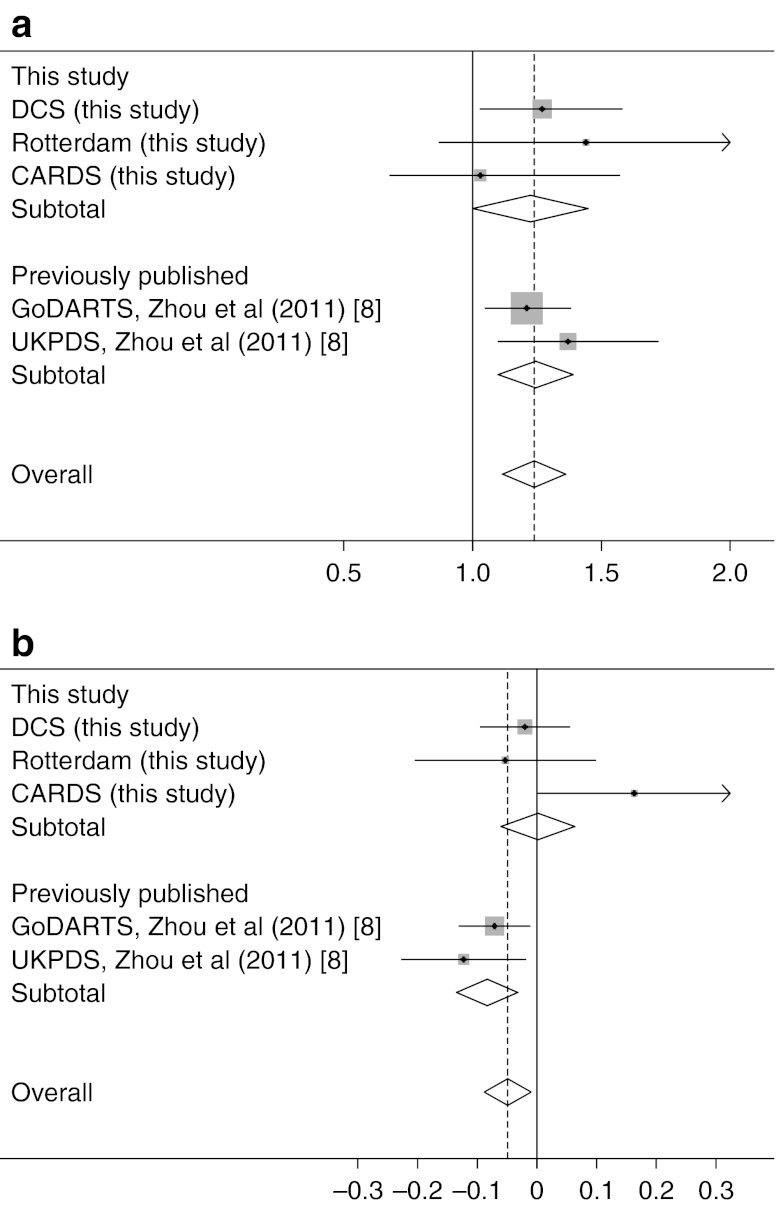
Association between rs11212617, metformin treatment response and treatment HbA_1c_ in different cohorts. The grey squares and horizontal lines indicate (**a**) the cohort-specific ORs and 95% CI for the ability to reach the treatment target of HbA_1c_ ≤7% (53 mmol/mol) and (**b**) the cohort-specific β-coefficients and 95% CI for the treatment HbA_1c_ as a continuous variable. The size of the squares is proportional to the weights of the studies. GoDARTS and UKPDS data were from Zhou et al [8]

In the meta-analysis of the three cohorts, there was an increase in the odds of treatment success with the presence of the C allele (OR 1.24, *p* = 0.016), in the whole group and where restricted to those starting metformin monotherapy alone (OR 1.38, *p* = 0.015). These results are consistent in size and direction with our previous publication, such that a combined meta-analysis including the GoDARTS and UKPDS stage 2 replication datasets from that publication resulted in a combined OR of 1.25 (95% CI 1.13, 1.38), *p* = 7.8 × 10^−6^; *I* ^2^ = 0.0%, *p* = 0.74; *p*_Harbord_ = 0.505 (Table 2, Fig. 1a, electronic supplementary material [ESM] Fig. 1). When the analysis was restricted to those with a baseline HbA_1c_ >7.0 % (53 mmol/mol), similar results were obtained (ESM Table 1). In the meta-analysis of the three novel cohorts where HbA_1c_ reduction was assessed as a quantitative trait, there was no significant effect of rs11212617 on HbA_1c_ reduction, partly because of the CARDS data being in the opposite direction to the other two cohorts, as reported previously (Fig. 1b). In the combined meta-analysis of all five replication cohorts from this and our previous study, the C allele was associated with an HbA_1c_ decrease of −0.050 (95 % CI −0.089, −0.010) per copy of the C allele (*p* = 0.013; *I* ^2^ = 59%, *p* = 0.05; *p*_Egger_ = 0.44; Table 3, Fig. 1b, ESM Fig. 1b). Again, when the analysis was restricted to those with a baseline HbA_1c_ above 7.0 % (53 mmol/mol), similar results were obtained (ESM Table 2).

## Discussion

In this study, we show replication of the reported association between a SNP at a locus that includes the *ATM* gene and glycaemic treatment success with metformin in the Dutch DCS cohort [8]. We noted a similar, although non-significant, trend in the smaller Rotterdam Study, but this trend was not observed in another small study, CARDS. Meta-analysis of data from these cohorts, which, despite intrinsic differences, provide clear, directionally consistent effects and shows replication of the previously reported association [8].

Importantly the effect of the SNP on metformin treatment success was observed in most cohorts, while there was no evidence for bias, and the characteristics of the cohorts differ, including data from population-based cohorts, prospective cohorts and clinical trials, indicating that the effect and its size are robust across European white populations. Furthermore, it is important to note that the association with treatment success is present regardless of baseline HbA_1c_, providing further evidence for the robustness of this observation.

The effect in the monotherapy group is larger than in the dual therapy group, as observed in the previous study. The underlying mechanism of this observation is unknown. As metformin is the recommended first-line therapy, patients receiving metformin monotherapy are probably earlier in their disease process. Accordingly, the observed difference between mono and dual therapy might be explained by the negative effects of prolonged disease duration on treatment efficacy. However, it might also be that sulfonylurea therapy antagonises some of metformin’s effects mediated through mTOR-dependent and -independent signalling, as has been suggested recently by Wang et al [16]. Further research is required to elucidate the exact underlying mechanism.

The effect of the SNP on the ability to achieve HbA_1c_ ≤7 % (53 mmol/mol) is stronger than the quantitative HbA_1c_ reduction, yet the quantitative trait should have greater statistical power. One possible explanation for the ‘treat-to-target’ model appearing to be better is that the patients and clinicians aiming for a particular target may not intensify treatment further once it is achieved. The low baseline HbA_1c_ of 6.7 % (50 mmol/mol) in the DCS probably reflects increased awareness of diabetes among patients and caregivers and current prescribing practice, where metformin is introduced immediately after diagnosis and based on fasting glucose rather than HbA_1c_ [17]. This most likely explains the lack of a genotype effect on HbA_1c_ reduction in this cohort, as there is not much scope for the absolute HbA_1c_ to decrease on metformin initiation.

We have shown, by additional replication and meta-analysis, that our initial report is consistent in multiple populations, supporting the finding that variation at the *ATM* locus is the most robust metformin response variant to date. Our data further support the notion that genetic variation does not only affect disease susceptibility, but also affects treatment response. Given the currently sparse efforts to elucidate the genetic architecture of type 2 diabetes treatment response, our data imply that future large-scale well-powered studies might be successful in identifying further novel loci affecting treatment response. This is particularly pertinent for metformin response, where the biology of its working mechanism is not fully elucidated. It is clear that additional research is required to establish that rs11212617 is the causal SNP and that the *ATM* gene is the causal gene at this locus. In addition, it is necessary to investigate the mechanism whereby *ATM* variation alters metformin response.

## Electronic supplementary material

Below is the link to the electronic supplementary material.

ESM Fig. 1 (PDF 39 kb)

ESM Table 1 PDF 8 kb

ESM Table 2 PDF 10.2 kb

## Abbreviations

AMPK: AMP-activated protein kinase
ATM: Ataxia telangiectasia mutated
CARDS: Collaborative Atorvastatin Diabetes Study
DCS: Diabetes Care System West-Friesland
DPP: Diabetes Prevention Program
GoDARTS: Genetics of Diabetes Audit and Research Tayside
HWE: Hardy–Weinberg equilibrium
LD: Linkage disequilibrium
SNP: Single-nucleotide polymorphism
UKPDS: UK Prospective Diabetes Study
