# Supplementary material for: A gene variant near ATM is significantly associated with metformin treatment response in type 2 diabetes: a replication and meta-analysis of five cohorts
Source: Diabetologia. 2012 Mar 28;55(7):1971–7. doi: 10.1007/s00125-012-2537-x (PMC3369131; doi:10.1007/s00125-012-2537-x)
Supplement: Supplementary file 1 — (PDF 39 kb) [file 125_2012_2537_MOESM1_ESM.pdf]

ESM Figure 1.

a.

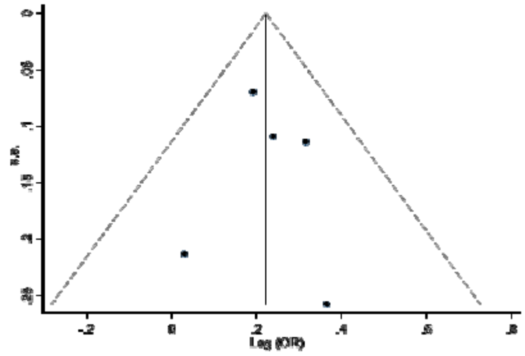

b.

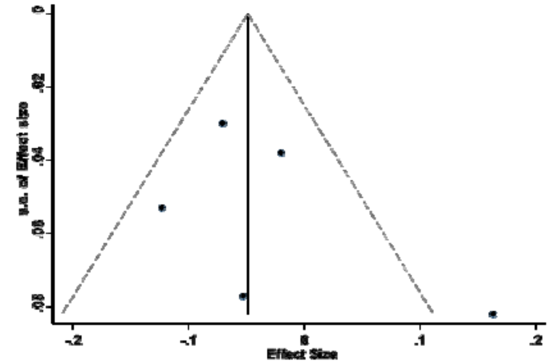

ESM Figure 1. Funnel plot with 95% confidence limits for a. the logistic regression meta-analysis (bias assessed with the Harbord method: 1.13 (95%CI -0.43, 0.53) p=0.505) and b. the linear regression meta-analysis (bias assessed with the Egger method: 1.76 (95% CI = -4.52, 8.04) p=0.438)
