# Supplementary material for: A gene variant near ATM is significantly associated with metformin treatment response in type 2 diabetes: a replication and meta-analysis of five cohorts
Source: Diabetologia. 2012 Mar 28;55(7):1971–7. doi: 10.1007/s00125-012-2537-x (PMC3369131; doi:10.1007/s00125-012-2537-x)
Supplement: Supplementary file 2 — PDF 8 kb [file 125_2012_2537_MOESM2_ESM.pdf]

ESM Table 1: Logistic regression for the ability to reach the metformin treatment target of HbA_{1c}<7% according to the *ATM* rs11212617 genotype. Only patients with a baseline HbA_{1c} >7% are included in the analysis.

Study	Group	n	OR (95%CI)	SE	P value
DCS	Total group	288	1.43 (1.02, 2.00)	0.17	0.041
	Mono therapy	136	1.79 (1.09, 2.93)	0.25	0.020
	Dual therapy	152	1.15 (0.70, 1.89)	0.25	0.57
Rotterdam Study	Total group	182	1.45 (0.87, 2.39)	0.26	0.15
	Mono therapy	65	1.97 (0.72, 5.42)	0.52	0.18
	Dual therapy	117	1.40 (0.77, 2.57)	0.31	0.27
CARDS	Total group	237	1.06 (0.69, 1.64)	0.22	0.77
	Mono therapy	71	1.56 (0.76, 3.10)	0.36	0.21
	Dual therapy	166	0.85 (0.47, 1.52)	0.30	0.59
Meta-analysis	Total group	707	1.31 (1.04, 1.66)	0.12	0.024
	Mono therapy	272	1.76 (1.20, 2.54)	0.19	0.0035
	Dual therapy	435	1.11 (0.81, 1.53)	0.16	0.52
Meta-analysis including stage 2 replication cohorts previously used by Zhou et al. (2011)					
GoDARTS	Total group	1783	1.21 (1.05, 1.38)	0.07	0.007
	Mono therapy	1291	1.29 (1.10, 1.51)	0.08	0.002
	Dual therapy	495	1.05 (0.81, 1.36)	0.13	0.70
UKPDS	Total group	799	1.24 (0.93, 1.66)	0.12	0.14
	Mono therapy	138	1.51 (0.89, 2.56)	0.27	0.13
	Dual therapy	661	1.14 (0.80, 1.62)	0.18	0.47
Meta-analysis	Total group	3289	1.24 (1.11, 1.38)	0.06	1.2*10-4
	Mono therapy	1701	1.36 (1.18, 1.56)	0.07	1.7*10-5
	Dual therapy	1591	1.09 (0.92, 1.30)	0.09	0.34

Additive logistic regression models were used for calculating the C-allelic odds ratios (OR) in each cohort. Covariates included where baseline HbA_{1c}, baseline gap (except DCS), daily dose, drug adherence (except for DCS and CARDS), and eGFR (except for CARDS and Rotterdam Study). In the meta-analysis a fixed effects model was used.
