# Supplementary material for: A gene variant near ATM is significantly associated with metformin treatment response in type 2 diabetes: a replication and meta-analysis of five cohorts
Source: Diabetologia. 2012 Mar 28;55(7):1971–7. doi: 10.1007/s00125-012-2537-x (PMC3369131; doi:10.1007/s00125-012-2537-x)
Supplement: Supplementary file 3 — PDF 10.2 kb [file 125_2012_2537_MOESM3_ESM.pdf]

ESM Table 2. Linear regression for treatment HbA_{1c} per ATM allele**.** Only patients with a baselineHbA_{1c} >7% are included in the analysis.

Study	Group	n	Beta (95%CI)	SE	P value
DCS	Total group	287	-0.060 (-0.231, 0.111)	0.087	0.49
	Mono therapy	135	-0.036 (-0.280, 0.208)	0.124	0.77
	Dual therapy	152	-0.085 (-0.335, 0.163)	0.126	0.50
Rotterdam	Total group	182	-0.053 (-0.205, 0.098)	0.077	0.49
Study	Mono therapy	65	-0.107 (-0.327, 0.113)	0.112	0.33
	Dual therapy	117	-0.069 (-0.266, 0.127)	0.100	0.49
CARDS	Total group	237	0.151 (-0.012, 0.332)	0.084	0.07
	Mono therapy	71	-0.120 (-0.465, 0.223)	0.173	0.49
	Dual therapy	166	0.270 (0.070, 0.469)	0.100	0.008
Meta-	Total group	706	0.010 (-0.083, 0.103)	0.048	0.83
analysis	Mono therapy	271	-0.084 (-0.231, 0.064)	0.075	0.27
	Dual therapy	435	0.058 (-0.063, -0.179)	0.004	0.35
Meta-analysis including stage 2 replication cohorts previously used by Zhou et al.(2011)					
GoDARTS	Total group	1783	-0.072 (-0.134, -0.011)	0.031	0.021
	Mono therapy	1291	-0.092 (-0.164, -0.019)	0.037	0.013
	Dual therapy	495	-0.040 (-0.160, 0.070)	0.059	0.45
UKPDS	Total group	799	-0.080 (-0.212, 0.053)	0.068	0.24
	Mono therapy	138	-0.272 (-0.564, 0.020)	0.149	0.07
	Dual therapy	661	-0.040 (-0.190, 0.107)	0.076	0.58
Meta-	Total group	3288	-0.051 (-0.099, -0.004)	0.024	0.035
analysis	Mono therapy	1700	-0.099 (-0.162, -0.035)	0.032	0.0023
	Dual therapy	1591	-0.005 (-0.077, 0.068)	0.037	0.89

An additive linear regression model was used for calculating the per C-allele change in treatment HbA_{1c}. Covariates included where baseline HbA_{1c}, baseline gap (except DCS), daily dose, drug adherence (except for DCS and CARDS), and eGFR (except for CARDS and Rotterdam Study). The p-value of the genotype effect is given. In the meta-analysis a fixed effects model was used**.**
